# Expression of human amyloid precursor protein in the skeletal muscles of *Drosophila *results in age- and activity-dependent muscle weakness

**DOI:** 10.1186/1472-6793-11-7

**Published:** 2011-04-25

**Authors:** Chul Kim, Sapeckshita Srivastava, Marian Rice, Tanja A Godenschwege, Brooke Bentley, Saranya Ravi, Shuang Shao, Craig T Woodard, Lawrence M Schwartz

**Affiliations:** 1 Molecular and Cellular Biology Program, University of Massachusetts, Amherst, MA 01003, USA; 2 Department of Biology, University of Massachusetts, Amherst, MA 01003, USA; 3 Department of Biological Sciences, Mount Holyoke College, South Hadley, MA 01075, USA; 4 Department of Biological Sciences, Florida Atlantic University, Boca Raton, FL 33431, USA; 5 Pioneer Valley Life Sciences Institute, 3601 Main Street, Springfield, MA, 01199, USA

**Keywords:** amyloid precursor protein (APP), Drosophila, muscle, mitochondria, electron microscopy, apoptosis, Parkin

## Abstract

**Background:**

One of the hallmarks of Alzheimer's disease, and several other degenerative disorders such as Inclusion Body Myositis, is the abnormal accumulation of amyloid precursor protein (APP) and its proteolytic amyloid peptides. To better understand the pathological consequences of inappropriate APP expression on developing tissues, we generated transgenic flies that express wild-type human APP in the skeletal muscles, and then performed anatomical, electrophysiological, and behavioral analysis of the adults.

**Results:**

We observed that neither muscle development nor animal longevity was compromised in these transgenic animals. However, human APP expressing adults developed age-dependent defects in both climbing and flying. We could advance or retard the onset of symptoms by rearing animals in vials with different surface properties, suggesting that human APP expression-mediated behavioral defects are influenced by muscle activity. Muscles from transgenic animals did not display protein aggregates or structural abnormalities at the light or transmission electron microscopic levels. In agreement with genetic studies performed with developing mammalian myoblasts, we observed that co-expression of the ubiquitin E3 ligase Parkin could ameliorate human APP-induced defects.

**Conclusions:**

These data suggest that: 1) ectopic expression of human APP in fruit flies leads to age- and activity-dependent behavioral defects without overt changes to muscle development or structure; 2) environmental influences can greatly alter the phenotypic consequences of human APP toxicity; and 3) genetic modifiers of APP-induced pathology can be identified and analyzed in this model.

## Background

Amyloid precursor protein (APP) is a type I glycotransmembrane protein with a large extracellular domain and a short cytoplasmic tail [reviewed in 1]. Its role in normal biological processes is poorly defined, but there is mounting evidence that it plays both autocrine and endocrine roles in neurite growth and enhanced memory function in mice [2,3].

APP became the subject of intense investigation when it was identified as a risk factor for Alzheimer's disease (AD) [4]. Individuals with an extra copy of the APP gene due to trisomy of chromosome 21 (Down Syndrome) also display early onset AD [5]. One point mutation in APP, referred to as the Swedish mutation, results in an early onset familial AD [6].

APP can be subjected to combinatorial cleavage by three different intramembrane secretases (α, β, and γ) to create a number of smaller peptides [7]. Cleavage by α-secretase, known as a non-amyloidogenic pathway, is responsible for a default secretory pathway and predominates in all non-neuronal cells. Cleavage by BACE (β-site APP cleaving enzyme) represents a minor pathway in most cell types, except for neurons. Like α-secretase cleavage of APP, the BACE-mediated fragment of APP undergoes further proteolysis by a γ-secretase complex to generate small peptides that typically range between 40-44 amino acids, although 46 amino acid fragments can be found in skeletal muscles. APP cleavage products are enriched in the brains of some AD patients [8], and exposure to the Aβ_42 _fragment is highly neurotoxic both *in vitro *and in *in vivo *animal models [9,10].

In addition to its well-documented roles in neurodegeneration, APP and its cleaved products have been also implicated in other diseases, most notably sporadic inclusion body myositis (s-IBM) [11], the most common skeletal muscle disorder of the elderly [12,13]. It has been reported that muscle biopsies from individuals with s-IBM contain Congo Red inclusions that are immunopositive for both APP [11] and Aβ_42 _[14]. However, this is not a universal observation [15], and proteomic analysis of IBM samples failed to reveal any APP proteolytic products [16]. In fact, a fundamental role for APP in the pathogenesis of s-IBM has been questioned recently [12,17,18].

While the role of APP in s-IBM awaits further analysis of clinical samples, it has been demonstrated that ectopic expression of either APP (either wild-type or Swedish mutant) or Aβ_42 _is sufficient to induce cell death in skeletal muscle either *in vitro *[19,20] or *in vivo *[21-23]. The biology of muscle makes it a much more tractable tissue to study than the central nervous system. Thus, studies designed to examine the effects of APP on muscle development and physiology may provide new insights into the general mechanisms that mediate APP induced pathogenesis.

To help define the mechanisms that could mediate APP toxicity in muscle, and to develop a genetic model for testing genetic and environmental interventions that might reduce APP-induced pathogenesis, we generated transgenic flies that express human APP in the skeletal muscles. We found that both genetic and environmental factors can interact to enhance or reduce APP-induced behavioral defects. These data suggest that the transgenic fly may represent a useful tool for defining the molecular mechanisms that mediate APP-induced muscle pathology and for identifying genetic and chemical modulators.

## Results and Discussion

### Muscle specific expression of human amyloid precursor protein (hAPP) induces an age-dependent reduction in climbing and flying activity

In order to study the role of human APP (hAPP) on the development and function of skeletal muscles, we took advantage of a transgenic fly line that expresses hAPP under the control of the Upstream Activating Sequence (UAS) [24]. These flies were crossed to a line that expresses the Gal4 transcription factor under the control of the muscle transcription factor *Dmef *[25], which expresses in all skeletal muscles and a few circadian neurons within the brain [26].

**Figure 1:**
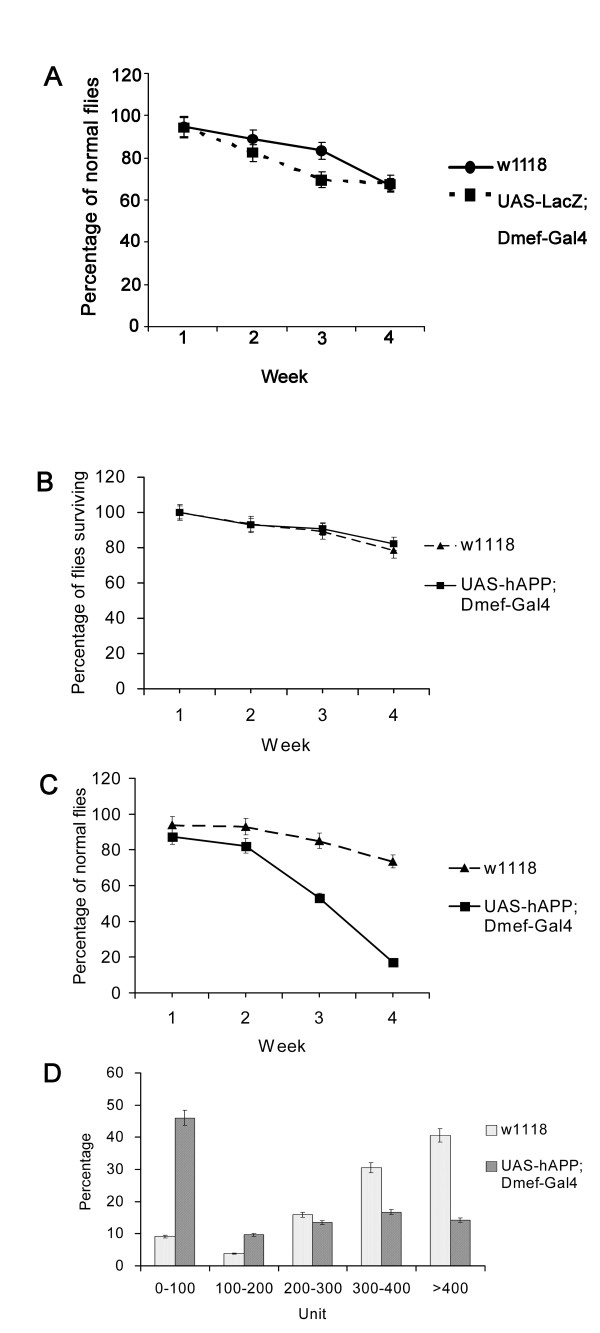
**hAPP-expressing flies display age-dependent defects in climbing and flying**. A. Climbing assays with wild type and LacZ expressing flies were performed every week. N = 136 and 85 for *w*^*1118 *^and LacZ expressing flies respectively. Mean +/- SEM. B. In order to measure the survival rates of flies, the numbers of *w*^*1118 *^and hAPP expressing transgenic flies were counted every week. Initially, 174 wild type and 166 transgenic flies were cultured. Mean +/- SEM. C. Climbing assays with *w*^*1118 *^and hAPP expressing transgenic flies in glass vials were conducted every week. N = 174 and 166 for wild-type and transgenic animals respectively. Mean +/- SEM. D. Flight assays with *w*^*1118 *^(light gray) and hAPP expressing flies (dark gray) were performed after five-week incubation in glass vials. Flies between 0-200 ml represent "poor fliers", while animals at 300-500 ml are designated as "good fliers". N = 131 and 90 for wild-type and transgenic animals respectively. Mean +/- SEM.

We employed several controls in each of these studies, including nontransgenic *w*^*1118 *^flies and transgenic flies expressing bacterial β-galactosidase (LacZ) under the control of Dmef-Gal4. The expression of LacZ both validated the anticipated expression of hAPP in muscles (data not shown) and controlled any effects caused by the competition of Gal4 promoter for general transcriptional machinery within the cells. Ectopic hAPP expressing flies (■) eclosed in normal numbers and displayed comparable longevity to the control lines (▲) suggesting that ectopic hAPP (■) was non-toxic (Figure 1B).

To assess the effects of ectopic hAPP on behavior, flies were tested for their ability to climb (Figure 1A). Both nontransgenic (*w*^*1118 *^●) and LacZ-expressing (■) flies displayed comparable levels of climbing activity during the one-month testing period. Transgenic flies expressing hAPP exhibited wild-type levels of climbing activity during the first two weeks of adulthood, but it declined during the subsequent weeks (Figure 1C) so that by the end of the fourth week, only about 20% of the transgenic flies could climb. In contrast, 75% of 1-month old wild-type *w*^*1118 *^control animals could climb. The climbing defect observed in transgenic flies was not due to a loss in their negative-geotropism, but rather, an apparent loss of strength that caused them to fall from the test cylinder before they could reach the test mark.

To evaluate flying behavior, animals were dropped into an oil-coated 500 ml graduated cylinder and then scored for their ability to remain near the top. Approximately 55% of the hAPP-expressing flies (dark gray) fell to the bottom 200 ml of the cylinder (poor fliers), while only about 12% of the controls (light gray) fell that distance (Figure 1D). Conversely, about 70% of the controls (light gray) remained within the top 200 ml (normal fliers), while only 20% of the transgenic flies (dark gray) were in this category. Taken together, the climbing and flying assays suggest that ectopic expression of hAPP results in an age-dependent defect in motor ability.

### Electrophysiological analysis of the neuromuscular junction

**Figure 2:**
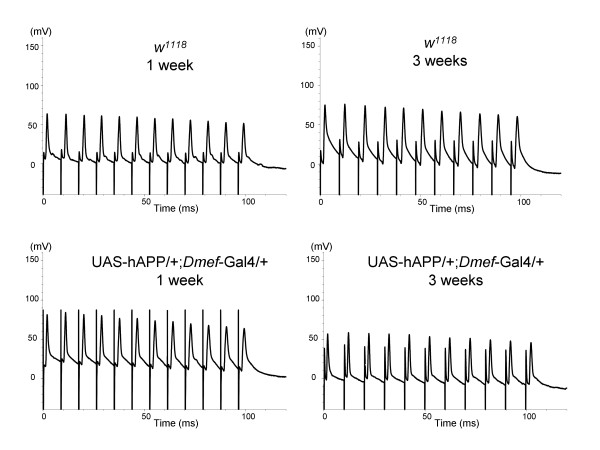
**Electrophysiological analysis of wild-type and hAPP-expressing flight muscles**. Sample traces of intracellular recordings of muscle responses from the DLFM following electrical stimulation of 1 week (A) and 3 week (B) old wild-type *w*^*1118 *^flies and 1 week (C) and 3 week (D) old *Dmef*-hAPP flies. No obvious differences were detected among the test groups independent of age or genotype.

Defects in motor function could reflect aberrations within the central nervous system, at the neuromuscular synapse, or within the muscle itself. To narrow our focus, we performed electrophysiological analyses of control and hAPP-expressing adult *Drosophila*. Intracellular glass recording microelectrodes were placed in the thoracic dorsal longitudinal flight muscle (DLFM) and tergotrochanteral motor muscle (TTM) of 1- and 3-week old adults, and the motor neurons were stimulated via the giant fibers using tungsten electrodes placed in the brain or directly in the thoracic ganglion. Both *w*^*1118 *^control and hAPP-expressing animals displayed normal DFLM muscle responses comprised of an evoked junction potential and a muscle action potential when repeatedly stimulated at 100Hz (Figure 2). There was no significant difference in amplitude size between animals recorded at 1 week versus animals recorded after 3 weeks (Student T-test, P < 0.05, Table 1). This suggests that motorneuron activation reliably results in muscle action potentials that should be sufficient to trigger a behavioral outcome mediated by muscle contraction. Consequently, the decay of climbing behavior in 3 week old hAPP expressing flies is not the result of a defect in motorneuron function and thus we focused on potential hAPP effects on the muscle.

**Table 1:** Amplitude sizes of recordings from the DLFM and the TTM

		DLFM	TTM
Genotype	N	mV/Stdv/SEM	mV/Stdv/SEM
1 week *w*^*1118*^	22	52.3/11.5/2.5	25.8/8.8/1.8
2 week *w*^*1118*^	22	53.3/18.0/3.8	23.2/7.7/1.7
1 week *Dmef/*hAPP	18	46.0/15.7/3.7	26.6/7.6/1.8
3 week *Dmef/*hAPP	22	45.0/12.4/2.6	23.2/6.8/2.0

Intracellular recordings were performed on the DLFM and TTM muscles following electrical stimulation as described in the Materials and Methods. Data are provided for both wild type *w*^*1118 *^and *Dmef*/hAPP flies at both 1 week and 3 weeks. Data is in mV and presented as the mean/standard deviation/standard error of the mean. N = number of flies tested for each condition. No significant differences between 1 week and 3 week old flies we observed (Student T-test, P < 0.05)

### Anatomical analysis of muscles

**Figure 3:**
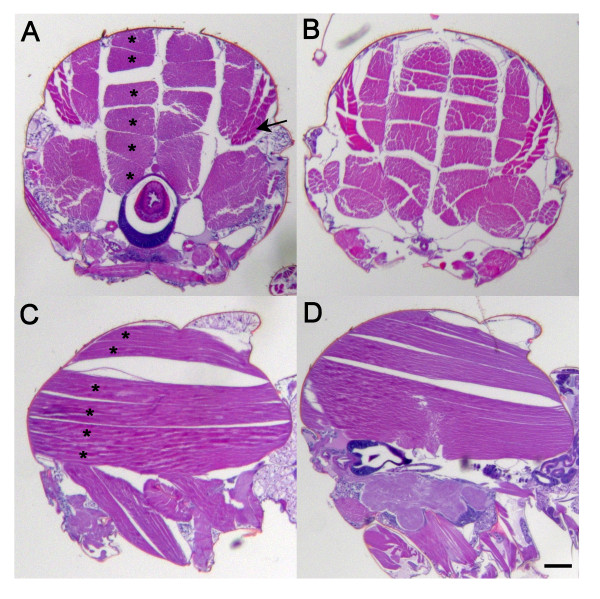
**Light microscopy of wild-type and hAPP-expressing flight muscles**. Age-matched transverse sections from three week old *w*^*1118 *^(A) and hAPP (B) expressing flies and age-matched sagittal sections from four week old *w*^*1118 *^(C) and hAPP (D) were conducted. Asterisks = dorsal longitudinal muscles, arrow = tergotrochanteral muscle. Scale bar = 0.1 mm.

To evaluate the effects of hAPP on muscle development and function, we performed both light and electron microscopy on the indirect flight muscles of control and hAPP-expressing flies. In Figure 3 we examined coronal (3 week old animals) and sagittal (4 week old animals) sections of the thorax to examine both the flight and leg muscles. At the light level, hematoxylin and eosin staining revealed well-developed muscles in both control (3A and 3C) and transgenic animals (3B and 3D), consistent with their ability to eclose, walk, climb, and fly as adults (Figure 1). Fiber number and diameter were comparable in wild-type and transgenic animals. These data support the hypothesis that ectopic expression of hAPP does not negatively impact myogenesis.

**Figure 4:**
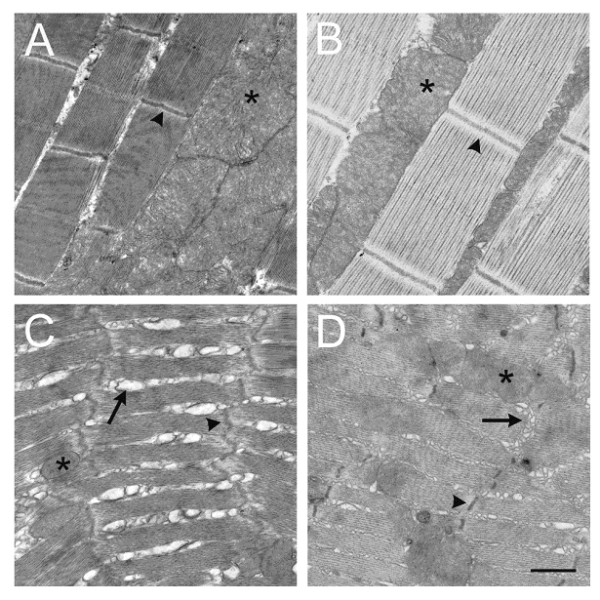
**Transmission electron microscopy of wild-type and hAPP-expressing flight muscle**. Dorsal longitudinal flight muscles (DLFM) from control (*w*^*1118*^, A) and hAPP (B) transgenic flies at three weeks of age. TTM muscles from control (*w*^*1118*^, C) and hAPP (D) transgenic animals at three weeks of age. Asterisks = mitochondria; Arrowheads = Z bands; Arrows = sarcoplasmic reticulum. Scale bar = 1 μm. Note that variations in the plane of section contribute to apparent changes in the sizes of some ultrastructural components.

We also performed transmission electron microscopy on the DLFM from 3-week old wild-type animals, which revealed well-developed Z-lines, M-lines, contractile apparatus and mitochondria (Figure 4A). Neither the muscle fibers themselves nor the internal membrane systems were swollen or disrupted. Anatomically, the DLFM of 3-week old transgenic animals were also grossly normal, and displayed well-defined sarcomeres (Figure 4B). As in the control muscles, dense rows of mitochondria were sandwiched between the bundles of contractile elements perpendicular to the Z bands. At the subcellular level, the organization of the TTM muscles differs from that of the DLFM both in terms of sarcomeric structure and the abundance of internal membrane systems of the T-tubule and sarcoplasmic reticulum (SR) (Figure 4C and 4D). At the electron microscopic level, 3 week old wild-type and hAPP transgenic TTM muscles were grossly indistinguishable (Figure 4C and 4D).

### Environmental control of muscle pathogenesis in hAPP-expressing flies

**Figure 5:**
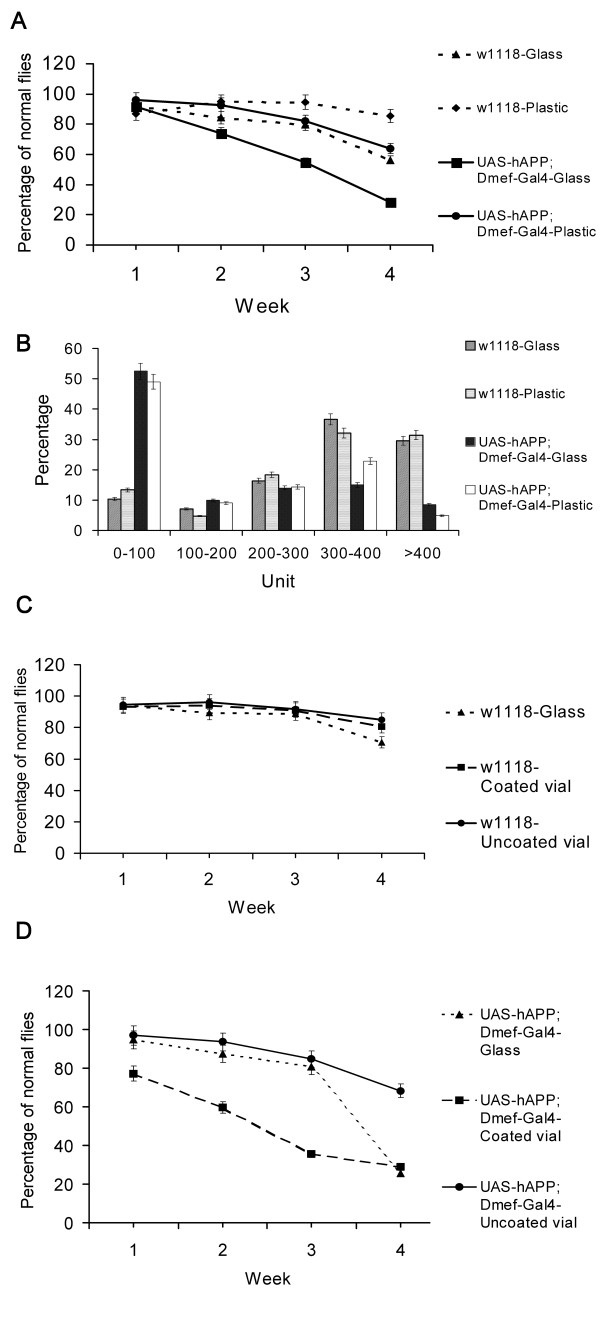
**Effect of vial type on climbing behavior in wild-type and hAPP transgenic flies**. A. The climbing ability of wild type and hAPP expressing flies reared in glass or plastic vials was determined. Percentage represents the portion of flies displaying normal behavior. N = 161 and 171 for wild type in plastic and glass vials, 171 and 173 for transgenic flies in plastic and glass vials, respectively. Mean +/- SEM. Climbing was statistically different by 4 weeks (p < 0.05). The error bars for the transgenic flies reared in glass vials were are not easily observed due to their small size. B. Flying assay was performed with 5 week old wild type flies cultured in glass (black) or plastic (white) vials and for hAPP expressing flies in glass (dark gray) or plastic vials (light gray). N = 199 and 126 for the wild-type animals reared in plastic and glass vials, and N = 126 and 128 for transgenic flies in plastic and glass vials, respectively. Mean +/- SEM. C. Climbing assays were performed with wild type flies cultured in glass, uncoated or siliconized plastic vials. In this set of experiments the flies reared in glass vials displayed a slightly better performance relative to the animals represented in Figure 5A. N = 117, 121, and118, respectively. Mean +/- SEM. Climbing was statistically different by 4 weeks (p < 0.05) D. Climbing assays were performed with hAPP expressing flies reared in glass, uncoated and siliconized plastic vials. N = 124, 118, and 141, respectively. Mean +/- SEM.

We performed many replicates of the climbing assay and consistently observed comparable age-dependent defects in transgenic animals. At one point in the study however, we switched from glass to polypropylene plastic vials for rearing the adults. While longevity was unchanged under these different rearing conditions (data not shown), the severity of the hAPP-induced climbing defect of flies cultured in the plastic vials was significantly reduced over the course of one month (Figure 5A). In order to verify that the rearing container impacted climbing behavior, cohorts of wild-type and hAPP-expressing animals were separated into glass or plastic vials and then subjected to the climbing assay. Wild-type animals displayed statistically improved climbing activity in plastic vials (♦) relative to glass vials (▲) (85% vs: 56% at week four; p < 0.05 ) (Figure 5A). A much more dramatic effect was observed for the hAPP-expressing animals. At 4 weeks 65% of plastic reared animals (●) could climb versus 25% for glass-reared animals (■) (p < 0.05). The benefits of plastic versus glass vials were restricted to the leg muscles and did not extend to the flight muscles (Figure 5B), since we did not observe any differences in flying activity between these two populations. These data suggest that only muscles that are actively challenged are the ones affected by the type of rearing substrate.

One possible mechanism for the differential effects of the vial composition on the development of muscle weakness is that the smoothness of glass relative to plastic requires more muscle strength to climb. To test this hypothesis, we siliconized plastic vials to make their surfaces smoother, and then tested new groups of control and transgenic animals. Survival was comparable in all three types of vials (data not shown). Wild type control flies climbed equally well in all three types of vials until week 3. After that, there was a small but statistically significant decline in climbing ability for flies reared in glass vials (▲) relative to coated (■) and uncoated plastic vials (●) (Figure 5C). However, the effects of vial type were much more dramatic in the hAPP transgenic flies (Figure 5D). Transgenic flies reared in siliconized plastic vials (■) displayed a rapid loss of climbing activity beginning even after the first week relative to those reared in glass (▲) or uncoated plastic vials (●). Between three and four weeks, there was a dramatic decline in climbing for hAPP transgenic flies reared in glass vials. These data suggest that the environmental factors (vial material) can have a significant impact on APP-induced pathogenesis.

### Coexpression of human Parkin rescues a hAPP-mediated climbing defect

**Figure 6:**
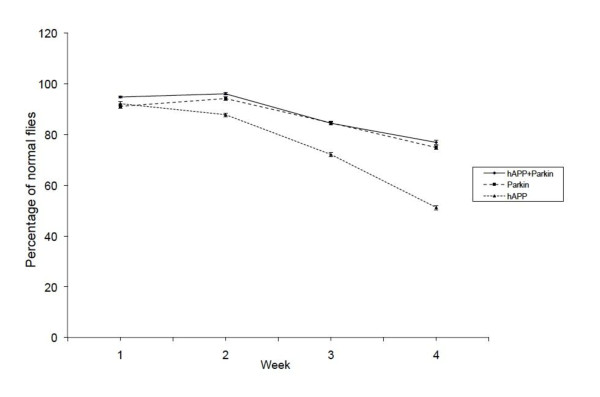
**Ectopic Parkin rescues hAPP-induced climbing defects in transgenic flies**. Climbing assays with transgenic flies expressing hAPP alone, hParkin alone, and hAPP plus hParkin in glass vials were conducted each week. N = 54 for all genotypes. Mean +/- SEM.

Mutations in the ubiquitin E3 ligase Parkin is the primary cause of Autosomal Recessive Juvenile Parkinsonism [27]. Loss of Parkin function endangers some cells, most notably midbrain dopaminergic neurons. However, *in vitro *studies with muscle cells have demonstrated that expression of ectopic Parkin can protect muscle from the toxic effects of accumulation of amyloid peptides [28]. To determine if this protein could also protect skeletal muscles *in vivo *from hAPP-induced damage, we generated transgenic flies that express human Parkin and/or hAPP in the muscles, and then monitored the ability of the adults to climb (Figure 6). Flies expressing hAPP (▲) demonstrated a significant decline in climbing ability beginning in the third week. Co-expression of Parkin (♦) rescued this defect and allowed the flies to climb at wild type levels (Figure 6).

APP has been the focus of intensive investigation for its possible role in human diseases, most notably Alzheimer's disease. Numerous studies have demonstrated that ectopic expression of APP, or its proteolytic products, most notably Aβ_42_, can trigger synapse loss and neuron death in both *in vitro *and *in vivo *models [9,10]. In fly, ectopic expression of Aβ_42 _or Aβ_40 _results in age-dependent neuron loss [29]. Interestingly, males were more severely impacted than females, although this may reflect differences in driver expression rather than differential gender sensitivity.

These same proteins have been proposed to accumulate within aggregates in patients with other diseases as well, such as inclusion body myositis [30], although this observation has been controversial [17,18]. Nevertheless, experimental studies have demonstrated that ectopic expression of either APP or Aβ_42 _is sufficient to induce muscle cell death both *in vitro *[19,20] and in transgenic mouse models [22,23,31]. In the nematode *Caenorhabditis elegans*, expression of Aβ_42 _results in protein aggregations within body wall muscles that result in paralysis and reduced longevity [32-34].

The transgenic fly model described in the present study complements and extends some of the data obtained with mouse and worm models, as well as some of the features of s-IBM. In s-IBM patients and animal models designed to simulate the disorder, individuals produce muscles that appear to be morphologically and physiologically normal, but develop progressive age-dependent muscle weakness in mid-to-late adulthood [12,22,32] (Figure 1C and 1D). This loss of muscle strength is not accompanied by detectable changes in neuromuscular synapse activity, suggesting that the defects arise within the muscles themselves. Indeed, we did not observe any changes in following frequency or spike amplitude in the muscles of our hAPP-expressing animals (Figure 2). These data would argue against a negative influence of hAPP expression in the small subset of circadian neurons in the brain that also express MEF2 [26]

The fly model described in this study does differ from both s-IBM and other hAPP transgenic mouse and worm models in that they did not produce protein aggregates within the sarcoplasm that can be detected with either Congo Red staining (data not shown) or ultrastructural analysis (Figure 4). It is possible that the level of hAPP expression was below the threshold required for macro-aggregation, or alternatively, that flies have intracellular mechanisms that limit aggregate formation. It has been noted that flies do not make inclusion bodies in the skeletal muscles with other aggregation prone proteins, like the polyglutamine-rich Huntington protein [35]. Nevertheless, the present fly model may provide insight into the molecular mechanisms that mediate hAPP induced pathology.

Several mechanisms have been proposed for the toxic effects of APP on skeletal muscle function including: defective regulation of ryanodine receptor-dependent sarcoplasmic Ca^2+ ^release [36], CD8+ cytotoxic T cell invasion [37], an autophagic mechanism [38], and myostatin activity [39].

The observation that ectopic expression of the ubiquitin E3 ligase Parkin can rescue the hAPP-associated defects in climbing in our transgenic model agrees well with *in vitro *results from other groups [16,27,40,41]. At least two possible Parkin associated mechanisms can ameliorate ectopic APP-induced behavioral defects. In mammalian muscle, Parkin prevents hAPP-induced muscle degeneration by inhibiting accumulation of toxic Aβs and also protects cells from mitochondrial-specific toxins like rotenone and carbonyl cyanide 3-chlorophenylhydrazone, but not from other toxins like calcium ionophore A23187 or H_2_O_2 _[28]. Loss of function mutations in the parkin gene result in age-dependent defects in flight muscle maintenance in fly and are also associated with mitochondrial defects [42]. Interestingly, these defects can be prevented by increased mitochondrial fission [43]. Taken together, these data support the hypothesis that Parkin may reduce mitochondrial oxidative stress or maintain respiratory functions to help preserve mitochondrial integrity.

We have demonstrated that environmental factors, such as rearing surface (glass versus plastic), have a dramatic effect on the timing and severity of hAPP-induced pathogenesis, suggesting a possible interplay between environmental and genetic factors. To our knowledge, this is the first report of any behavioral activities that can be tied to the material used for rearing flies. The simple manipulation of changing the rearing vessel may represent a valuable tool for genetic screens in fly designed to identify work-associated genes in fly muscle.

Only limited data exist on the role of exercise in the progression of myopathies in human [44,45]. Interestingly, Arnardottir *et al. *[44] have suggested that moderate exercise may retard the symptoms of s-IBM. Rearing flies in plastic vials greatly reduced the timing and severity of symptoms relative to rearing in glass (Figure 5A). Since the only difference between animals reared in glass versus plastic was the nature of the vessel, we speculated that surface properties accounted for the observed effects. Glass vials have a smoother surface than plastic and therefore it is presumably harder for the fly to climb. This extra work would increase the mechanical stress on the muscle fibers and might contribute to damage or generation of reactive oxygen species (ROS) by mitochondria. We tried to test this hypothesis directly by setting up devices that would force the animal to walk more, such as placing the vials on a slowly moving rocking table. Unfortunately, these efforts to voluntarily increase motor activity did not appear to alter animal behavior, so instead we changed the surface properties of the plastic vials by siliconizing them. This subtle manipulation had a profound effect on the time course and severity of the hAPP-induced abnormal activities. This effect was specific to the muscles that were forced to work, since this treatment did not alter flying behavior.

## Conclusions

In this study, we generate a transgenic *Drosophila *model that targets the expression of hAPP to the skeletal muscles in order to understand its effect on muscle development and degeneration in the adult. Ectopic expression of hAPP causes age- and activity-dependent muscle weakness without concomitant structural damage that can be discerned at either the light or electron microscopic levels. The effects of ectopic hAPP can be ameliorated by hParkin expression. Taken together, these data suggest that in *Drosophila*, hAPP-induced muscle deficits are mediated by not only genetic, but also environmental factors, and that muscle work/stress may contribute to pathogenesis. This animal model is useful to identify additional genetic and environmental modulators and understand underlying mechanisms.

## Methods

### Generation of transgenic UAS-human Parkin Drosophila lines

In order to generate UAS-human Parkin, pRK5-myc-Parkin generously provided by Dr. Dawson [46] and a pUAST vector were digested with *EcoR*I and *Not*I restriction enzymes (Promega) and ligated at 16°C overnight with T4 ligase (Promega). The construct, pUAST-myc-human Parkin was sent to the Transgenic *Drosophila *Fly Core (Charlestown, MA) for embryonic injections. Transgenic lines were selected and cultured at room temperature.

### Drosophila

Transgenic flies expressing human APP under the control of the Gal4 upstream activating sequence (UAS) were provided by Dr. Kalpana White [24]. A *Dmef*-GAL4 line driving the expression Gal4 in mesodermal precursors [25] was provided by Dr. Leo Pallanck. Progenies from these crosses were maintained at 25°C with 12 hour-light/dark cycle. Both males and females were used in each experiment.

### Climbing Assay and flight assay

Flies were divided into 5-7 groups of 25 flies each and reared in standard glass or plastic vials (Genesee Scientific) as noted. In some experiments plastic vials were siliconized with glass cleaner (Rain-X). The vials were exchanged every two to three days and animals were scored weekly in the climbing assay. Briefly, after a 1 minute acclimation, the percentage of flies that could climb past the 30ml mark in a 50ml cylinder within 30 seconds was scored as successful. Trials were performed 3 times/vial. After five weeks, the flies were subjected to the flying test. The animals were dropped into a 500ml graduate cylinder coated with mineral oil. The number of immobilized flies was determined for each 100 ml section of the cylinder.

### Statistical Analysis

Statistical significance was determined using Student's T test and one-way-analysis of variances (ANOVA) using GraphPad Prism software (GraphPad Software, La Jolla, CA). Data were considered statistically significant when their p values were 0.05 or less.

### Electrophysiology

Standard methods were used to get intracellular recordings from the dorsal longitudinal flight muscle (DLFM) and the tergotrochanteral muscle (TTM) [47]. Flies were anesthetized and waxed, ventral side down, onto a small podium in a Petri dish. The motorneurons were either indirectly stimulated via the Giant Fiber (GF) in the brain or directly with thoracic stimulation using two etched tungsten electrodes and by providing pulses of 40-60V for 0.03ms using a Grass S44 stimulator (Grass Instruments). A tungsten electrode placed in the abdominal cavity served as a ground. Glass electrodes pulled to a resistance of 40-60MW were filled with saline and were driven through the cuticle into the DLFM and TTM muscle fibers. Signals were amplified, digitized and analyzed with pClamp 10 software to monitor amplitude size and the ability of the muscle membrane to follow multiple stimuli at 100HZ (Getting Instruments and Molecular Devices).

### Microscopy

Flies were collected at different ages, the heads and abdomens removed, and the carcass immersed in 4% paraformaldehyde overnight at 4°C. They were then dehydrated, embedded in paraffin and sectioned at 10 μm followed by staining with hematoxylin and eosin for light microscopy. For transmission electron microscopy, flies were prepared as previously described [48,49]. A fly was placed thorax facing upward and fully covered in a droplet of Optimal Cutting Temperature (OCT) compound on a glass microscope slide. The fly was snap frozen in liquid nitrogen (-196°C) and then gently fractured with a liquid nitrogen-chilled razor blade struck with a hammer to longitudinally bisect the flies. Care was taken to ensure that the droplet of tissue was completely submerged in liquid nitrogen throughout the fracturing process, so that at no time was any sample exposed to air and that the razor blade contacted the OCT compound, not the thorax itself. The split pieces were immediately transferred to a container of primary fixative made of 2.5% glutaraldehyde in 0.1 M phosphate buffer at pH 7.4, and allowed to thaw and fix overnight at 4°C. The fixative was changed several times to ensure its concentration was not altered by the thawing OCT. After rinsing in 0.1 M NaPO_4 _buffer at pH 7.4, the tissue was post-fixed in 2% osmium tetroxide in 0.1 M NaPO_4 _buffer at pH 7.4 for one hour. The sample was then rinsed in distilled water and stained with 1% uranyl acetate for 30 minutes. It was then rinsed in water and dehydrated through an ethanol series from 30% to 100%. The sample was then infiltrated with Spurr's extra low viscosity resin, and after embedding, mounted fracture-surface up and thin-sectioned with a diamond knife for TEM analysis. Multiple sections from at least eight different flies of each genotype and age were examined for each experiment.

## List of Abreviations

**hAPP**: human amyloid precursor protein; **AD**: Azheimer's disease; **s-IBM**: sporadic inclusion body myositis; **A**β: β amyloid peptide; **UAS**: upstream activating sequence; **LacZ**: β-galactosidase; **Dmef**: *Drosophila *myocyte enhancer factor; **GF**: Giant Fiber; **DLFM**: dorsal longitudinal flight muscle; **TTMn**: tergotrochanteral motorneuron; **TTM**: tergotrochanteral muscle; **SR**: sarcoplasmic reticulum; **ROS**: reactive oxygen species.

## Competing interests

The authors declare that they have no competing interests.

## Authors' contributions

CK generated the transgenic animals, performed most of the behavioral assays and analyses, and co-wrote the manuscript. SS performed some behavioral assays as part of her honors thesis work. MR and SS prepared the samples and conducted transmission electron microscopic analysis (as part of SS's honors thesis). TG performed the electrophysiological studies. BB performed the light microscopy studies. SR and CTW performed the analyses of the hParkin expressing flies. CTW and LS designed, discussed and helped co-write the manuscript with CK. All authors read and approved the final manuscript.
